# A Bayesian approach to Mendelian randomization with multiple pleiotropic variants

**DOI:** 10.1093/biostatistics/kxy027

**Published:** 2018-08-01

**Authors:** Carlo Berzuini, Hui Guo, Stephen Burgess, Luisa Bernardinelli

**Affiliations:** 1 Centre for Biostatistics, The University of Manchester, Jean McFarlane Building, University Place, Oxford Road, Manchester M13 9PL, UK; 2 Department of Public Health and Primary Care, University of Cambridge, Cambridge, UK and MRC Biostatistics Unit, University of Cambridge, Cambridge, UK; 3 Department of Brain and Behavioral Sciences, University of Pavia, Pavia, Italy

**Keywords:** Correlated instruments, Egger regression, Instrumental variable, Mediation, Median estimator, Metabolomics, Shrinkage, Sparsity prior

## Abstract

We propose a Bayesian approach to Mendelian randomization (MR), where instruments are allowed to exert pleiotropic (i.e. not mediated by the exposure) effects on the outcome. By having these effects represented in the model by unknown parameters, and by imposing a shrinkage prior distribution that assumes an unspecified subset of the effects to be zero, we obtain a proper posterior distribution for the causal effect of interest. This posterior can be sampled via Markov chain Monte Carlo methods of inference to obtain point and interval estimates. The model priors require a minimal input from the user. We explore the performance of our method by means of a simulation experiment. Our results show that the method is reasonably robust to the presence of directional pleiotropy and moderate correlation between the instruments. One section of the article elaborates the model to deal with two exposures, and illustrates the possibility of using MR to estimate direct and indirect effects in this situation. A main objective of the article is to create a basis for developments in MR that exploit the potential offered by a Bayesian approach to the problem, in relation with the possibility of incorporating external information in the prior, handling multiple sources of uncertainty, and flexibly elaborating the basic model.

## 1. Introduction

Many statistical studies aim to assess the causal effect of a phenotype or exposure ($X$) on an outcome ($Y$). In many such studies, an experimental design is unfeasible, and the only remaining option is to work on the basis of observational data. Unfortunately in this case, no matter how impeccable is the study design, how accurate are the observations and smart the inference algorithm, there is no guarantee that the result will not be biased due to unobserved confounding or reverse causation. A useful approach to this situation is Mendelian randomization (MR) (Katan, 1986; Davey Smith and Ebrahim, 2003; Lawlor *and others*, 2008). The bare bones of the idea are that, under certain assumptions, for a phenotype $X$ to be a causal influence on an outcome $Y$, we expect a genetic variant $Z$ that modulates $X$ to likewise affect $Y$. Information about $Z$ can then be used as an instrument to assess the causal effect of $X$ on $Y$, despite confounding.

The potential impact of MR on science cannot be underestimated (Robinson *and others*, 2017). In various occasions, an MR study based on observational data has predicted the outcome of a clinical trial, thereby supporting or casting doubt on the motivating causal hypothesis (Voight *and others*, 2012). Furthermore, MR can help biologists reconstruct a disease process from its molecular causes to its phenotypic manifestations, and unravel causal relationships of pharmacological relevance through the analysis of biobank data.

Early implementations of MR used a single or a handful of instrumental variants, under the untestable assumption that these variants are not pleiotropic, i.e. that they affect the outcome only through the changes they induce in the exposure. Recent developments, mostly in the frequentist realm, have focused on methods that use multiple instruments, while allowing for “cryptic” pleiotropy, i.e. allowing an unspecified subset of the instruments to affect the outcome directly. Examples of multi-instrument Mendelian randomization (MIMR) methods that allow for cryptic pleiotropy are the Egger regression (ER) and the weighted median estimator (WME) method of Bowden *and others* (2016).

The existing frequentist approaches to MR do not coherently account for important sources of uncertainty, such as the uncertainty arising from the estimation of the instrument-exposure (i-e) associations. Hence our concern that these methods may yield over-optimistic results. We attempt to remedy this by proposing a Bayesian approach to MR (see Didelez and Sheehan, 2007; Didelez and Sheehan, 2008; Burgess and Thompson, 2010; Burgess and Thompson, 2012; Jones *and others*, 2012), which deals with cryptic pleiotropy and can in principle acknowledge uncertainty at all levels of the model. Our method allows the user to shape the prior on the basis of external information, for stronger and more accurate inferences, although it will work with vague prior specifications, too. It combines the power of Bayesian analysis with that of Markov chain Monte Carlo (MCMC) inference, for an exceptional freedom in elaborating the basic model. Extensions of our method to deal with non-linear dependencies and model uncertainty are under investigation, but remain outside the scope of this article. We restrict our attention to continuous $X$ and $Y$ variables.

In Section 2, we review past related work, and place our method in that context. In Section 3, we introduce our MIMR framework in a simple setting. The idea here is that the pleiotropic effects are represented in the model by unknown parameters, with an independence sparsity prior that assumes an unspecified subset of these parameters to be zero. Incorporating this prior yields a proper posterior for the causal effect, which we MCMC-sample to obtain point and interval estimates. Also discussed in this section is the use of external information to shape an informative prior. In Section 4, we assess the performance of our method in relation to the number of instruments, the amount and direction of pleiotropy, and the degree of linkage disequilibrium (LD) between the instrumental variants, taking the performance of the WME method as a reference. Thanks to the explicit modeling of the direct instrument-outcome (i-o) effects, our approach bears relationships with mediation analysis. This connection is explored in depth in Section 5, where we consider a problem involving two exposures (instead of one), and use our method to estimate direct and indirect effects. This is further illustrated in Section 6 with the aid of a study in metabolomics. This article is based on the decision-theoretic causality framework proposed by Dawid (2000) and described in Chapter 4 of Berzuini *and others* (2012).

## 2. Background

Let $U$ denote a set of imperfectly observed exposure-outcome (e-o) confounders, responsible for the correlation between $X$ and $Y$ being not totally attributable to a causal relationship. In order for a scalar variable $Z$ to qualify as an instrument for estimating the causal effect of $X$ on $Y$, we generally require it to satisfy the following three conditions, where we use the notation $A \,\perp\!\!\!\perp\, B \mid C$ for “$A$ is independent of $B$ given $C$” (Dawid, 1979), and $A \,\not\!\perp\!\!\!\perp\, B \mid C$ for the negation of the same sentence:

Condition 1 (marginal relevance) $Z$ is associated with the exposure, formally $Z \,\not\!\perp\!\!\!\perp\, X$.

Condition 2 (confounder independence) $Z$ is independent of the e-o confounders, formally $Z \,\perp\!\!\!\perp\, U$.

Condition 3 (exclusion restriction) $Z$ is independent of $Y$, given $U$ and $X$, formally $Z \,\perp\!\!\!\perp\, Y \mid (U,X)$.

**Fig. 1.**
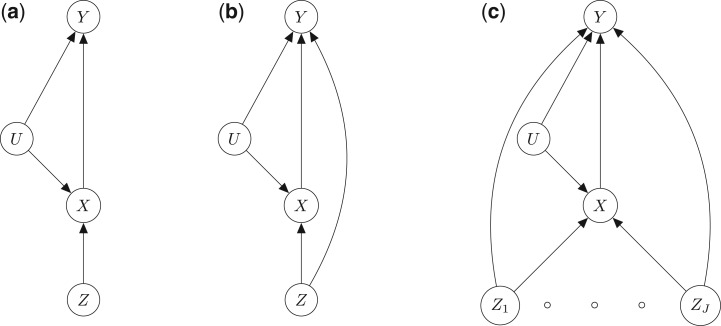
Conditional independence graph representations of a Mendelian randomization problem. In (a) the graph represents a set of conditional independence assumptions that do not violate Conditions 1–3 of Section 2. In (b), the arrow from $Z$ to $Y$ violates Condition 3. In (c) the graph represents a class of problems with multiple instruments, where Conditions 1 and 2 are not violated.

The last two conditions are not testable on the basis of the usually available $(X,Y,Z)$ data. Three examples of MR problem are graphically represented in Figure 1, where the $X \rightarrow Y$ arrow represents the causal effect of inferential interest, the $Z \rightarrow Y$ arrow a pleiotropic effect, and a $Z \rightarrow X$ arrow an i-e association, which none of the methods discussed assumes to be causal. We regard the graphs of Figure 1 as expressing sets of conditional independence relationships, which can be read off them with the aid of the $d$-separation criterion of Geiger *and others* (1990). Conditions 1–3 are satisfied in Figure 1a. Condition 3 is violated in Figure 1b by the presence of the $Z \rightarrow Y$ arrow.

With reference to Figure 1a, if we assume linear additive dependencies between the variables in the graph, and let $\hat{\beta}_{Y}$ and $\hat{\beta}_{X}$ denote the estimated slopes in the regressions of $Y$ on $Z$ and $X$ on $Z$, respectively, then the instrumental variable (IV) estimator of the causal effect of $X$ on $Y$ is $\hat{\beta}_{Y}/\hat{\beta}_{X}$. A small sample size and/or weak i-e associations may cause the data to deviate from Condition 2 (Nelson and Startz, 1990), and consequently the IV estimate to be affected by the so-called weak instruments bias.

Existing frequentist methods admit a collection of independent instruments, $Z_1, \ldots , Z_J$, and they require Conditions 1 and 2 to hold for all instruments, formally $Z_j \,\not\!\perp\!\!\!\perp\, X$ and $Z_j \,\perp\!\!\!\perp\, U$, for $j= 1, \ldots , J$, as in Figure 1c. In these methods, each $j$th instrument contributes a separate IV estimate $\hat{\beta}_{Yj}/\hat{\beta}_{Xj}$ of the causal effect of $X$ on $Y$. When the IV estimates of several instruments show reasonable concordance, it would appear that a causal conclusion is defensible, pleiotropy notwithstanding. This idea is developed by Egger *and others* (1997), who suggest that concordance can be tested by regressing $\hat{\beta}_{Yj}$ on $\hat{\beta}_{Xj}$. Under the assumption that the i-e associations (or instrument strengths) are independent of the direct effects (pleiotropic associations), usually referred to as the INSIDE assumption (Kolesar *and others*, 2014), evidence of a linear relationship between $\hat{\beta}_{Yj}$ and $\hat{\beta}_{Xj}$ will support (and provide an estimate of) the causal effect of interest, whether or not the instruments satisfy Condition 3. For finite numbers of instruments, the frequentist interpretation of the INSIDE is that the correlation between pleiotropic and i-e associations is zero. This is an untestable property, although some indirect empirical evidence (Pickrell *and others*, 2015) can be summoned in its support. The Egger method requires the instrumental SNPs to be recoded to ensure that the i-e associations have the same sign, although, unfortunately, INSIDE is sensitive to changes such. Moreover, by treating the $\{\hat{\beta}_{Xj}\}$ as fixed quantities, the Egger method ignores the imprecision introduced by their estimation.

Another popular approach to MIMR is the median estimator. If Conditions 1 and 2 are valid, the instruments are independent and at least half of them satisfy Condition 3, then the median of their corresponding IV estimates will be a consistent estimate of the causal effect (Han, 2008). Bowden *and others* (2016) proposed a widely used weighted version of this estimator—the WME of the causal effect.

In this article, we propose a Bayesian approach to MR that allows an unspecified subset of the instruments to be pleiotropic, provided that Condition 2 and a Bayesian version of the INSIDE assumption (see Condition 4 in the next section) are satisfied. The proposed approach has the following distinguishing features. It allows for (moderate) instrument–instrument correlation, and does not require the signs of the instrument effects to be manipulated. It treats the i-e associations as random quantities, which we can learn about via prior-to-posterior updating. Once the posterior distributions (e.g. for the i-e associations and for the causal effect, etc.) have been calculated, they can be used as priors in future studies, in what can be regarded as a sequential learning process. Finally, while the aforementioned frequentist methods emphasize the construction of estimators for specific situations, our combined use of Bayesian inference and MCMC computation allows the researcher to focus on model choice, to better explore the possibility of tackling elaborated versions of the basic model.

We conclude this section with a note on the decision-theoretic formulation of causality proposed by Dawid (2000), and on the corresponding definition of causal effect, which we adopt in the present work. In accord with this formulation, we define the causal effect of $X$ on $Y$ as the difference between the expected values of $Y$ under a (hypothetical) intervention that imposes on $X$ a reference value $x_0$ and another intervention that imposes a generic value $x$. To express this, let the symbol $F_X$ label the regime under which the value of $X$ is generated, with $F_X=a$ indicating that $X$ is fixed to value $a$ by an intervention of the relevant type, and $F_X=\emptyset$ denoting the observational regime under which the data have actually been obtained. Then the average causal effect (ACE) of $X$ on the continuous outcome $Y$ is defined by $ACE = E(Y \mid F_X =x)-E(Y \mid F_X =x_0)$. Based on our observational data (obtained exclusively under regime $F_X = \emptyset$) we can estimate ACE under the (bold) assumption $Y \,\perp\!\!\!\perp\, F_X \mid (X,U)$, that the conditional distribution of $Y$ given $X=x$ in the generic individual characterized by a specific value of $U$, depends on $x$, but not further on whether the value $x$ has arisen by passive observation or through the intervention of interest. The implications of this condition in a MR context, and, more in general, in the context of IV analysis, are examined in Chapter 4 of Berzuini *and others* (2012)

## 3. Methods

We shall now introduce our approach to MR with reference to a one-sample setting, where each individual is characterized by a complete set of observed values for $X$, $Y$ and $Z_1, \ldots , Z_J$. We assume linear additive dependencies and write

(3.1) \begin{eqnarray*}\label{full1}P(U) &=& N(0,1),\\\end{eqnarray*}

(3.2) \begin{eqnarray*}\label{full2}P(X \mid Z_1, \ldots , Z_J, U) &=& N(\omega_X +\sum_{j=1}^J \alpha_j Z_j +\delta_X U, \sigma_X^2),\\\end{eqnarray*}

(3.3) \begin{eqnarray*}\label{full3}P(Y \mid X, Z_1, \ldots , Z_J, U) &=& N(\omega_Y + \theta X +\sum_{j=1}^J \beta_j Z_j +\delta_Y U,\sigma_Y^2),\end{eqnarray*}

where $N(a,b)$ stands for a normal distribution with mean $a$ and variance $b$, the symbol $\alpha \equiv (\alpha_1, \ldots , \alpha_J)$ denotes the i-e associations and $\beta \equiv (\beta_1, \ldots , \beta_J)$ are the pleiotropic effects. The causal effect of interest, denoted as $\theta$, represents the change in $Y$ caused by an interventional unit change in $X$. We may equivalently write

(3.4) \begin{eqnarray*}\label{triangular1}P(X \mid Z_1, \ldots , Z_J) &=& \omega_X +\sum_{j=1}^J \alpha_j Z_j +A,\\\label{triangular2}\end{eqnarray*}

(3.5) \begin{eqnarray*}P(Y \mid X, Z_1, \ldots , Z_J) &=& \omega_Y + \theta X +\sum_{j=1}^J \beta_j Z_j +B,\end{eqnarray*}

with $A \sim N(0, \delta_X^2+\sigma_X^2)$, $B \sim N(0, \delta_Y^2+\sigma_Y^2)$ and $cov(A,B) = \delta_X \delta_Y$. Equations (3.4– 3.5) involve a vector of parameters $\Phi \in R^{(2J+6)}$, with $\Phi \equiv (\omega_X,\omega_Y, \tau_X \equiv \sqrt(\delta_X^2+\sigma_X^2), \tau_Y \equiv \sqrt(\delta_Y^2+\sigma_Y^2), \lambda \equiv \delta_X \delta_Y, \beta,\alpha,\theta)$. The model is not completely identifiable, in the sense that the information contained in the observed covariances does not lead to a unique solution for $\Phi$ or any subset of $\Phi$ containing the parameter of inferential interest, $\theta$. In fact, parameters $(\omega_X, \alpha, \tau_X)$*are* identified by the $J+2$ conditions provided by equalities $E(X \mid Z_1, \ldots , Z_J) = \omega_X + \sum_{j=1}^J \alpha_j Z_j$ and $var(X \mid Z_1, \ldots , Z_J) = \tau_X^2$. Unfortunately, the remaining $J+4$ parameters, including the causal effect of interest, $\theta$, remain unidentified. This is because the equality $E(Y \mid X, Z_1, \ldots , Z_J) = \omega^{'}_Y + \theta^{'} X + \sum_{j=1}^J \beta^{'}_j Z_j$ (with $\omega^{'}_Y = \omega_Y - \omega_X \frac{\lambda}{\tau_X^2}, \theta^{'} = \theta + \frac{\lambda}{\tau_X^2}$ and $\beta^{'} = \beta - \alpha \frac{\lambda}{\tau_X^2}$) provides additional $J+2$ conditions, and a further condition is obtained from the equation $var(Y \mid X,Z_1, \ldots , Z_J) = \tau_Y^2- \frac{\lambda^2}{ \tau_X^2}$, for a total of additional $J+3$ conditions, which are not sufficient to identify $J+4$ parameters.

From a Bayesian point of view, non-identifiability can be negotiated by using a scientifically plausible prior that induces a proper posterior on $\theta$. Formally, if $D$ denotes data, the posterior can always be written in the product form:

$$\begin{eqnarray*}P(\omega, \tau, \lambda,\theta,\alpha, \beta \mid D) =P(\omega_X,\alpha,\tau_X \mid D) \;P(\omega_Y,\theta, \tau_Y,\lambda \mid \beta,\omega_X,\alpha,\tau_X, D)\;P(\beta \mid \omega_X,\alpha,\tau_X, D).\end{eqnarray*}$$

Because the last term above is the conditional posterior of an unidentifiable parameter, it reduces to the conditional prior: $P(\beta \mid \omega_X,\alpha,\tau_X, D)= P(\beta \mid \omega_X,\alpha,\tau_X)$, which leads to

$$\begin{equation*}P(\omega, \tau, \lambda,\theta,\alpha,\beta \mid D) = P(\omega_X,\alpha,\tau_X \mid D)\; P(\omega_Y,\theta, \tau_Y, \lambda \mid\beta, \omega_X, \alpha,\tau_X, D)\;P(\beta \mid \omega_X,\alpha,\tau_X),\end{equation*}$$

from which it follows that we may make the full posterior distribution proper by allowing the last term of the above product to take the form of a proper distribution. To proceed, we introduce the following Bayesian interpretation and generalization of INSIDE:

Condition 4 (instrument effects orthogonality (IEO)) Each component of $\beta$ is a priori independent of the parameters of the exposure model, $P(\beta \mid \omega_X,\alpha,\tau_X) = P(\beta)$, and we specify a proper and scientifically plausible prior $P(\beta)$. One option is to impose $\beta =0$, as in a standard IV analysis, which however will often be unrealistic. A second option is to impose that the effect exerted by each instrument on the outcome through the mediation of $X$ is greater in magnitude than the corresponding pleiotropic (unmediated) effect. We use none of these. In the following section, we construct $P(\beta)$ from our belief that some of the components of $\beta$ are zero.

### 3.1. The prior

We shall now discuss the prior specifications for model (3.1–3.3). In many applications, it will be reasonable to assume that some components of vector $\beta$ are zero, i.e. that an unspecified subset of the set of instruments have no pleiotropic effect. This justifies imposing on $\beta$ a shrinkage prior, e.g. by taking each $\beta_j$ to be a priori independently drawn from a Laplace (double exponential) distribution with mean $0$ and unknown variance $2 \tau^2$, with $\tau$ distributed a priori as $Cauchy^{+}(0,1)$, where $Cauchy^{+}(0,a)$ denotes the half-Cauchy density on the positive reals, with scale parameter $a$. An alternative choice is to impose on each $j$th component of $\beta$ the horseshoe shrinkage prior proposed by Carvalho *and others* (2010), which has the hierarchical structure $p(\beta_j \mid \phi_j) = N(0, \phi_j^2), \;\;p(\phi_j \mid \gamma) = Cauchy^{+}(0,\gamma), \;\; p(\gamma) = Cauchy^{+}(0,1 )$, where the degree of shrinking of each $j$th component of $\beta$ is controlled by an unknown parameter $\phi_j$. A high value of $\phi_j$ corresponds to a near-zero value of the shrinkage weight, $\kappa_j = 1/(1+ \phi_j^2)$, in which case this prior leaves the magnitude of $\beta_j$ almost unaffected. In contrast, a near-zero value of $\phi_j$ corresponds to a near-unit shrinkage weight, which will result in the estimate of $\beta_j$ being heavily shrunk towards zero. Under the horseshoe prior, each $\beta_j$ is mixed over its own $\phi_j$, with $\phi_j$ drawn from a $Cauchy^{+}(0,\gamma)$ distribution governed by an unknown parameter $\gamma$. Both the $\phi_j$ parameters, which are in charge of controlling the local degrees of shrinking, and parameter $\gamma$, which controls the global degree of shrinking, are inferred from the data, with minimal input from the user. With $\gamma=1$, the horseshoe specifications induce on $\kappa_j$ a horseshoe-shaped $Beta(.5,.5)$ distribution with one peak at $\kappa_j=0$ and another at $\kappa_j=1$. The two peaks may be interpreted in terms of the horseshoe prior inducing sparsity in a selective fashion. The lower peak of the distribution of $\kappa_j$ accounts for the small components of $\beta$, which our model recognizes as noise and heavily shrinks towards zero. The upper peak of the distribution accounts for the large components of $\beta$, which our model recognizes as pleiotropic, and leaves almost unaffected, thereby reducing the influence of the pleiotropic instruments on the estimate of $\theta$.

In our experience, assigning the remaining parameters uniform priors does not cause numerical problems, thanks to the ability of the Stan toolbox (STAN Development Team, 2014) to determine a sensible bounding of the search space via variational algorithms. However, we shall often wish to make our priors informative, for stronger inferences. In future studies, we speculate that it will be possible to shape informative priors on the basis of data collected in previous studies (provided these satisfy the necessary conditions). For example, $(X, Z)$ data from past studies can be used to construct a prior for $(\alpha, \omega_x, \tau_x)$, in such a way to reduce the weak instruments bias.

Consider also that mathematical relationships between parameters may be used to derive sensible local priors. For example, parameters $\delta_X$ and $\sigma_X$ are not identifiable, but the model links them to $\tau_X$ through the identity $\tau_X^2 = \delta_X^2+\sigma_X^2$, which justifies the inequalities $\delta_X \le \tau_X$ and $\sigma_X \le \tau_X$. Because we are able to learn about $\tau_X$ from external data, we can use this information, in conjunction with the above inequalities, to derive joint prior bounds for $\delta_X$ and $\sigma_X$ (not illustrated in this article). Alternatively, we may establish an upper bound for $\tau_X^2$, denoted by $u_{\tau_X^2}$, and impose the prior bound $\delta_X^2+\sigma_X^2 \le u_{\tau_X^2}$. In some situations, a posterior distribution for the causal effect might become available from previous studies, and be used, under assumptions, as our prior for $\theta$. Prior information about $\beta$ might become available with the development of web repositories containing lists of instruments for specific exposures. Finally, in certain situations it might be reasonable to assume a priori that each direct effect $\beta_j$ is smaller in magnitude than the corresponding indirect effect, $\alpha_j \theta$.

In our analyses of real and simulated data, we assigned $\sigma_X$ and $\sigma_Y$ uniform prior distributions with positive support. We assigned $\theta, \delta_X$, $\delta_Y$, $\omega_X$ and $\omega_Y$ independent uniform priors, and we took each $\alpha_j$, for $j=1, \ldots , J$, to be independently drawn from a normal $N(\mu_{\alpha}, \sigma_{\alpha}^2)$ prior, with hyperparameters $\mu_{\alpha}$ and $\sigma_{\alpha}$ subject to uniform priors.

## 4. Simulation experiment

We performed a simulation experiment to evaluate our model’s performance in relation to the number of instruments and individuals, the direction and amount of pleiotropy, and the degree of correlation between the instruments. Although performance comparisons are not a primary objective of this article, we shall compare our method’s performance with the WME in terms of bias, coverage and power.

Our simulations were based on sequences of SNPs of real individuals, with each SNP expressed on an interval scale as an allele dose (0,1,2). We considered the 21 simulation scenarios described in Table 1. In each of these, we simulated 800 datasets with the causal effect $\theta$ set to zero, and further 800 datasets with $\theta$ set to 0.35, which allowed us to assess each method’s performance under the null and under the alternative hypothesis. The SNP sequences changed from one individual to the next, but they were kept fixed across scenarios and simulations, except for scenarios 14 to 21, where they changed from one scenario to the next to represent different degrees of LD between the SNPs.

**Table 1.** Comparative assessment of the proposed method (with a horseshoe prior for $\beta$) and of the WME, in relation to the mean pleiotropy, the number of instruments, the degree of linkage disequilibrium ($R^2$) between instruments and the dispersion of the $\alpha$ instrument-exposure associations (column 4)

Scenario	Number of individuals	Mean pleiotropy	Standard deviation $\alpha$	No. of instruments	Linkage disequilibrium ($R^2$)	Coverage under the null	Coverage under the alternative	Power	Bias under the null	Bias under the alternative	Coverage under the null	Coverage under the alternative	Power	Bias under the null	Bias under the alternative
					Our method					Weighted median estimator							
1	500	0.012	0.02	60	0	79	86	93	$-$0.04	$-$0.06	70	78	88	$-$0.04	$-$0.04
2	500	0.006	0.02	60	0	89	86	95	$-$0.02	$-$0.03	78	79	92	0.01	$-$0.03
3	500	0	0.02	60	0	91	94	99	$-$0.003	0.02	80	81	94	0.01	0.01
4	500	$-$0.006	0.02	60	0	90	88	99	0.03	0.03	75	80	98	0.04	0.06
5	500	$-$0.012	0.02	60	0	85	81	99	0.06	0.06	73	73	98	0.07	0.08
6	500	0.012	0.02	20	0	90	88	62	$-$0.03	0.02	82	80	60	$-$0.02	0.08
7	500	0	0.02	20	0	90	93	73	0.01	0.01	80	87	71	0.03	0.01
8	500	$-$0.012	0.02	20	0	86	89	80	0.06	0.09	81	82	78	0.07	0.12
9	500	0.012	1.0	60	0	95	94	99	0.00	0.00	83	97	99	0.00	0.00
10	500	0.006	1.0	60	0	96	94	99	0.00	0.00	89	98	99	0.00	0.00
11	500	0	1.0	60	0	96	95	99	0.00	0.00	90	99	99	0.00	0.00
12	500	$-$0.006	1.0	60	0	95	94	99	0.00	0.00	88	99	99	0.00	0.00
13	500	$-$0.012	1.0	60	0	95	93	99	0.00	0.00	85	99	99	0.00	0.00
14	500	($-$0.012 to 0.012)	0.02	60	0.33	96	96	97	0.05	0.04	12	11	98	0.09	0.08
15	500	($-$0.012 to 0.012)	0.02	60	0.54	96	95	94	$-$0.05	$-$0.06	12	12	98	0.07	0.1
16	500	($-$0.012 to 0.012)	0.02	60	0.63	81	70	95	0.09	0.09	8	10	97	0.13	0.11
17	500	($-$0.012 to 0.012)	0.02	60	0.70	82	72	94	0.07	0.08	2	3	96	0.08	0.09
18	300	($-$0.012 to 0.012)	0.02	60	0.33	98	96	72	$-$0.05	$-$0.05	19	24	93	$-$0.14	$-$0.13
19	300	($-$0.012 to 0.012)	0.02	60	0.53	98	96	66	$-$0.04	$-$0.04	11	12	85	$-$0.17	$-$0.16
20	300	($-$0.012 to 0.012)	0.02	60	0.62	96	95	48	0.06	0.07	8	10	84	$-$0.18	$-$0.17
21	300	($-$0.012 to 0.012)	0.02	60	0.70	85	90	12	$-$0.15	$-$0.18	4	9	83	$-$0.19	$-$0.17

Coverage and power are expressed as percentages.

Each of Scenarios 1 to 13 uses independent SNPs, and is characterized by (i) the sample size reported in column 2 of Table 1, (ii) the value of $\mu_{\beta}$, the mean pleiotropic effect, reported in column 3, and (iii) the value of $\sigma_{\alpha}$ reported in column 4, which controls the variability of the strength, $\alpha$, from one instrument to the next. Note that by varying $\mu_{\beta}$, we explore different types of pleiotropy: balanced ($\mu_{\beta}=0$), negative ($\mu_{\beta} < 0$) and positive ($\mu_{\beta} > 0$). In particular, by allowing $\mu_{\beta}$ to take values $\pm 0.012$, we have included situations where the pleiotropic component of the effect of the instrument on the outcome is on average stronger than the component mediated by the exposure (indirect component). At each new simulation, new values for the model parameters were generated. In particular, in Scenarios 1 to 13, each component of $\alpha$ was independently drawn from $N(-0.07, \sigma_{\alpha}^2)$. A randomly selected subset (40%) of the components of $\beta$ were independently drawn from $N(\mu_{\beta}, 0.05^2)$, the remaining components being set to 0. The proportion of instruments with a significant ($P < 0.05$) marginal association with the exposure varied between 70% and 100% across the simulations. Also, parameters $\omega_X$ and $\omega_Y$ were drawn from $N(3.3, 0.2^2)$ and $N(0.9, 0.2^2)$, respectively, and $\delta_X$ and $\delta_Y$ were independently drawn from $N(-0.1, 0.02^2)$, so as to have a positive average correlation between $X$-errors and $Y$-errors. Parameters $\sigma_X$ and $\sigma_Y$ were sampled from sharp inverse-gamma distributions with means 0.1 and 0.3, respectively. Conditional on the generated parameter values, at each new simulation we generated values for variables $U, X, Y$, for each individual, on the basis of Equations (3.1–3.3) and in conformity with the IEO condition.

Scenarios 14 to 21 involve instrumental SNPs with increasing degrees of mutual correlation (average $R^2$ reported in column 6). These scenarios were generated in the same way as the previous ones, except for vector $\alpha$. After the elements of this vector were simulated, the majority of them were set to zero, so as to mimic the situation where only a small number of instruments have a non-null causal or conditional effect on the exposure. Also, the components of $\beta$ were independently drawn from $N(\phi, 0.05^2)$, with $\phi$ uniformly distributed between $\pm 0.012$, so as to embrace situations where the pleiotropic effect is on average stronger than the i-o indirect effect.

Each simulated dataset was analyzed via WME to obtain a point and a bootstrapped 95% confidence interval for $\theta$, and then via our model (with a horseshoe prior for $\beta$) to obtain a posterior mean and a 95% credible interval for $\theta$. On the basis of these results, we assessed performance in terms of bias, coverage and power. The analysis with our model was performed by using the Hamiltonian MCMC methods (Metropolis *and others*, 1953; Neal, 2011) provided by the program Stan (STAN Development Team, 2014). Stan employs a combination of variational (Wainwright and Jordan, 2008) and MCMC methods. The former are used to generate an approximation of the posterior distribution of the model parameters. The approximation is then used to guide the MCMC exploration of the posterior. No major Markov chain mixing problems were encountered.

We shall now briefly discuss the results of the simulations. Scenarios 1 to 8 were based on independent instruments. Table 1 tells us that, in these scenarios, (i) in both methods an increase in the number of instruments corresponds to an increase in power, (ii) in both methods an increase in the number of instruments corresponds to a drop in coverage under the null, the drop being modulated by the amount of directional pleiotropy, and (iii) in both methods, positive pleiotropy reduces power. In our case, a positive pleiotropy corresponds to the direct and indirect effects of the instruments’ effects on the outcome having on average the opposite sign.

A comparison between the results of Scenarios 1–5 and Scenarios 9–13, all of which involve independent instruments, suggests that in both methods a higher value of $\sigma_{\alpha}$, which means a higher number of strong instruments, improves power and coverage under the null. In our method, this was sufficient to bring coverage under the null into the nominal range. This did not happen with WME, although in Scenarios 9–13 WME slightly outperforms our method in terms of coverage under the alternative.

In Scenarios 14 to 17, and in both methods, the progressively increasing degree of LD between SNPs causes a marked drop in coverage and a slight drop in power. In the presence of LD, the gap in performance between the two methods is dramatic. This is unsurprising, because WME was developed with independent instruments in mind. This pattern is confirmed in Scenarios 18 to 21, where, in addition, we observe the effect of reducing the number of individuals from 500 to 300. The reduction makes power more vulnerable to presence of LD between the instruments.

Our method appears to outperform WME in terms of coverage under the null (in all scenarios), and in terms of power (in all scenarios with independent instruments).

## 5. Incorporating mediation

This section extends our approach to deal with two (instead of one) exposures or intermediate phenotypes, $X_1$ and $X_2$. Within this more general setting, we shall use our method to estimate direct and indirect effects, and to combine, albeit under strong parametric assumptions, the capabilities of MR and mediation analysis.

**Fig. 2.**
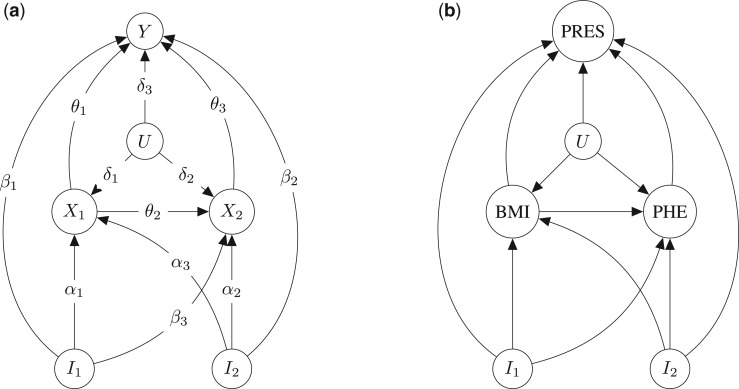
(a) Graphical model for the class of problems discussed in Section 5, (b) Application of the graphical model to our study in Section 6.

Figure 2a portrays a problem where $X_1$ is a putative cause of $X_2$. Suppose, we accept the assumptions represented in the figure. Suppose, we are interested in the direct causal effect of $X_1$ on $Y$ (controlling for $X_2$), and in the indirect effect of $X_1$ on $Y$ (via $X_2$). Suppose, we are also interested in the causal effects of $X_1$ on $X_2$ and of $X_2$ on $Y$. Let the set of instruments, $Z_1, \ldots , Z_J$, consist of two non-overlapping subsets, $I_1$ and $I_2$, with $I_1 \equiv (Z_1, \ldots , Z_{J_1})$ and $I_2 \equiv (Z_{J_1+1}, \ldots , Z_{J_1+J_2})$, with $J=J_1+J_2$. Assume for simplicity that $I_1 \,\perp\!\!\!\perp\, I_2$. Let $Z_{-j} \equiv (Z_{1}, \ldots , Z_{j-1}, Z_{j+1}, \ldots ,Z_{J})$. We elaborate (3.1–3.3) into:

(5.1) \begin{eqnarray*}\label{mediation}P(X_1 \mid I_1, I_2, U) &=& N(\omega_1 +\sum_{j=1}^{J_1}\alpha_{1j} Z_j+ \sum_{j=1}^{J_2} \alpha_{3j} Z_{J_1+j}+ \delta_1 U, \sigma_1^2),\\P(X_2 \mid X_1, I_1, I_2, U) &=&N(\omega_2 + \theta_2 X_1 +\sum_{j=1}^{J_1} \beta_{3j} Z_j+ \sum_{j=1}^{J_2} \alpha_{2j} Z_{J_1+j}+ \delta_2 U, \sigma_2^2),\nonumber \\P(Y \mid X_1, X_2, I_1, I_2, U) &=&N(\omega_Y + \theta_1 X_1+ \theta_3 X_2 +\sum_{j=1}^{J_1} \beta_{1j} Z_j+ \sum_{j=1}^{J_2} \beta_{2j} Z_{J_1+j}+ \delta_3 U,\sigma_Y^2),\nonumber\end{eqnarray*}

with $U \sim N(0,1)$, $\alpha_{1} \equiv (\alpha_{1j}, \ldots , \alpha_{1J_1})$, $\alpha_{2} \equiv (\alpha_{2j}, \ldots , \alpha_{2J_2})$, $\alpha_{3} \equiv (\alpha_{3j}, \ldots , \alpha_{3J_2})$, $\beta_{3} \equiv (\beta_{3j}, \ldots , \beta_{3J_1})$. The causal effect of $X_1$ on $X_2$ is represented by parameter $\theta_2$, whereas the direct causal effect of $X_1$ on $Y$ (controlling for $X_2$) is represented by parameter $\theta_1$, and the indirect causal effect of $X_1$ on $Y$ is represented by $\theta_2 \theta_3$. The model equations satisfy Condition 2. When all components of $\alpha_1$ and $\alpha_2$ differ from zero, they satisfy also:

Condition 5 (sequential relevance) Each component of $I_1$ is associated with $X_1$, conditional on the remaining instruments, and each component of $I_2$ is associated with $X_2$, conditional on $X_1$ and the remaining instruments. This condition is formally expressed by $Z_{j} \,\not\!\perp\!\!\!\perp\, X_{1} \mid Z_{-j}$ for $j=1, \ldots , J_1$, and $Z_{j} \,\not\!\perp\!\!\!\perp\, X_{2} \mid (Z_{-j},X_1)$, for $j=J_1+1, \ldots , J$.

In the following, we show that, under the above conditions, and in the special case where $\beta_1= \beta_2 = \beta_3 =0$, all the parameters of model (5.1) and, in particular, the causal effects $(\theta_1, \theta_2, \theta_3)$, are identified.

First, we need to introduce the concept of “unblocked” path of a causal diagram. A path ($=$ sequence of adjacent edges) in a causal diagram is said to be *unblocked* if it involves one or more *colliders* (Geiger *and others*, 1990), i.e., if at least one pair of arrows point to a common node, $\rightarrow \circ \leftarrow $ (not the most general definition, but sufficient for our purposes).

We are now ready to show that $(\theta_1,\theta_2,\theta_3$) are identifiable, provided *(i)*$\alpha_1 \ne 0$, *(ii)*$\alpha_2 \ne 0$ and *(iii)*$\beta_1 =\beta_2 =\beta_3 =0$. To see this, consider Figure 2a in the simple case where $I_1$ and $I_2$ are scalar. Assume all variables represented in the graph have zero mean. Then the correlation $\rho_{AB}$ between two nodes of the graph, $A$ and $B$ say, is given by a sum of terms over all the unblocked paths that connect $A$ and $B$, with each term of the sum consisting of the product of the effects along the path (Wright, 1934). By using Figure 2a and Condition *(iii)*, we obtain $\rho_{X_1I_1} = \alpha_1 $ and $\rho_{X_1I_2} = \alpha_3$. The two equalities uniquely identify parameters $\alpha_1$ and $\alpha_3$, conditional on which we may then consider the system formed by equations $\rho_{I_1X_2} = \alpha_1\theta_2$ and $\rho_{I_2X_2} = \alpha_2 + \alpha_3 \theta_2$, which can be solved for $(\alpha_2, \theta_2)$ by virtue of Condition *(i)*, as its determinant does not vanish. This means that causal parameter $\theta_2$ is identified. Next, note that, under Condition *(iii)*, nodes $Y$ and $I_1$ are connected via two unblocked paths, and $Y$ and $I_2$ via further three unblocked paths, which leads to the two equations $\rho_{I_1Y} = \alpha_1\; \theta_1 +\alpha_1 \theta_2 \theta_3$ and $\rho_{I_2Y} = \alpha_3 \theta_1 + ( \alpha_3 \theta_2 + \alpha_2)\; \theta_3$. These can be solved for $\theta_1$ and $\theta_3$, conditional on the identifiable parameters $(\alpha_1,\alpha_2,\alpha_3, \theta_2)$, because Conditions *(i)* and *(ii)* prevent the determinant of the system, $\alpha_1\alpha_2$, from vanishing, which completes the proof.

We deal with the more general situation where the $\beta$-vectors depart from zero in the same way as in Section 3.1, i.e. by imposing on each of these vectors a sparsity prior that makes the posterior distribution of the causal effects proper. Simple averages of the MCMC samples generated from this posterior will give simulation-consistent point and interval estimates for any function of interest of parameters $(\theta_1, \theta_2, \theta_3)$, such as, for example, the indirect effect $\theta_2 \theta_3$ exerted by $X_1$ on $Y$. This is illustrated in the next section.

## 6. Illustrative application

Past decades have witnessed an unprecedented worldwide rise in obesity. Excess body fat, as measured by body mass index (BMI $=$ weight in kilograms divided by the square of the height in meters) is a major risk factor for cardiovascular disease (CVD), among other disorders. The increased incidence of CVD associated with adiposity is believed to be mediated both by abnormalities in carbohydrate metabolism and by an increase in blood pressure. As far as the latter is concerned, various authors have found evidence of BMI being a causal factor for hypertension, and in this section, we shall corroborate this hypothesis by applying our MR approach to data from the general population, by using a recently proposed measure of blood pressure burden defined as the sum of the diastolic and systolic arterial pressures, hereafter, denoted as PRES (Nair, 2016). Part of our analysis is motivated by recent metabolite profiling studies, that have highlighted deviations in molecular signatures of BMI. Many of these studies compared small groups of individuals with large differences in adiposity, and it remains unclear whether those deviations are also observed in the general population. One putative molecular signature of obesity is the $\alpha$-aminoacid phenylalanine (PHE) (Jones, 1996; Droyvold *and others*, 2005; Shah *and others*, 2012; Moore *and others*, 2014; Wuertz *and others*, 2015; Hao *and others*, 2016). Recent research also highlights PHE as a putative mediator of the causal effect of body fat on blood pressure.

In the following analysis, we shall put these hypotheses under scrutiny by using our MR approach. We shall first use MR to assess the putative causal effect of BMI on PHE. In a subsequent stage of the analysis, we shall assess the causal effect of BMI on PRES, in terms of a direct causal effect, and of an indirect causal effect mediated by PHE.

We analyzed a dataset of 520 unrelated individuals (aged 25–74) from a population-based Finnish cohort—the DILGOM (Dietary, Lifestyle and Genetic determinants of Obesity and Metabolic Syndrome) study (Inouye *and others*, 2010). Each individual in this study had serum metabonome information, a genome-wide genetic profile and measures of BMI, blood pressure and sex. The eighty instruments used in the analysis, $Z_1, \ldots , Z_{80}$, were SNPs with a significant ($P \le 10^{-5}$) BMI marginal association, and in negligible LD ($R^2 < 0.05$). These SNPs we treated as counts $(0,1,2)$ of minor alleles at the corresponding locus. Let $X$ (the exposure) represent the logarithm of BMI. Let $Y$ represent the log-concentration of PHE, and $W$ take value $0$ for female and $1$ for male.

WME gave an estimated causal effect of log BMI on log PHE of 0.25, with a 95% confidence interval of 0.18–0.31. Our analysis based on model (3.1–3.3) gave a posterior mean of 0.3, with a 95% credible interval of 0.19–0.42, representing a higher degree of uncertainty about the causal effect with respect to the WME estimate.

A number of studies (Kaplan *and others*, 2014, see) stress the differential prognostic significance of BMI across genders. This motivated our interest in incorporating an interaction between the effects of sex and BMI on the outcome. Recall that sex is denoted as $W$, with $W=0$ indicating female, and $W=1$ male. For purposes of illustration, we made the following simplifying assumptions. First, we assumed that sex is independent of the confounders $U$. Second, we assumed the effect of sex on either BMI or PHE not to interact with the effect of the instrumental variants on the same variable. The latter assumption is delicate, which invites caution in the interpretation of the results. To include the interaction, we extended model (3.1–3.3) as follows:

(6.1) \begin{eqnarray*}\label{fullinteraction}P(X \mid Z_1, \ldots , Z_{80}, U, W) &=& N(\omega_X +\sum_{j=1}^{80} \alpha_j Z_j+ \psi_{XW} W + \delta_X U, \sigma_X^2),\\P(Y \mid X, Z_1, \ldots , Z_{80}, U, W) &=& N(\omega_Y+ (\theta+\psi_{YXW} W)\; X + \sum_{j=1}^{80}\beta_j Z_j + \psi_{YW} W+ \delta_Y U,\sigma_Y^2), \nonumber\end{eqnarray*}

with $U \sim N(0,1)$. The causal effects of log BMI on log PHE are represented in the model equations by $\theta$ (in the females) and $\theta+\psi_{YXW}$ (in the males), with $\psi_{YXW}$ representing the interaction between sex and BMI. We used a horseshoe prior for $\beta$, and uniform priors for the remaining parameters. We ran 10 000 iterations of a Markov chain, and used the values generated during the second half of the chain to compute the estimates. Parameter $\psi_{YXW}$ had a posterior mean of $-0.14$ and a 95% credible interval of $-0.28$ to $-0.0062$, representing fair evidence of an interaction between BMI and sex in their causal effects on PHE. The causal effect of log BMI on log PHE had a posterior mean of 0.34 with a 95% credible interval of 0.21–0.47 in the females, and a posterior mean of 0.2 with a 95% credible interval of 0.098–0.3 in the males.

**Fig. 3.**
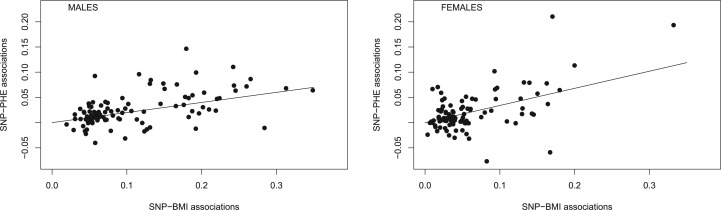
With reference to our analysis of Section 6, each $j$th instrument is represented in each of these plots by a black dot with co-ordinates ($\hat{\beta}_{Xj}, \hat{\beta}_{Yj})$ (see Section 2 for a definition of these symbols), as obtained from an analysis of the male (left plot) and female (right plot) individuals in the sample. The slope of the regression line is the Egger regression estimate of the causal effect.

In the scatter diagrams of Figure 3, each instrumental SNP is represented by a black dot with $x$-coordinate (respectively, $y$-coordinate) given by the coefficient of the least-squares regression of log BMI (respectively, log PHE) on that SNP, as obtained from an analysis of the male (left plot) and female (right plot) subsamples. The linearity of the relationship in both plots provides visual evidence of a causal effect of BMI on PHE, whereas the difference between the two slopes provides evidence of that causal effect interacting with sex.

**Fig. 4.**
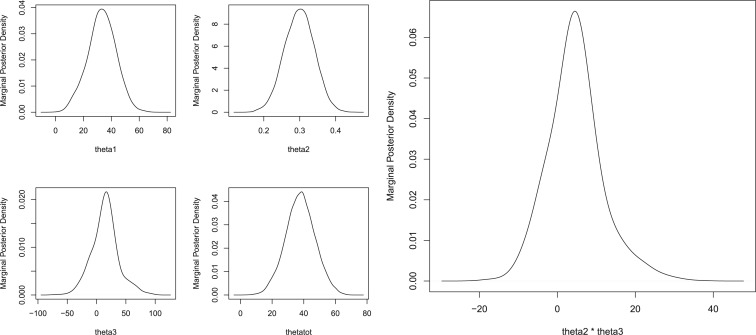
This figure summarizes results from our analysis of the illustrative problem of Section 6. Shown in this figure are the posterior distributions for key parameters of model (5.1), as obtained by applying the model to the DILGOM data, under the assumptions of Figure 2b. Parameter $\theta_1$ represents the controlled direct effect of BMI on PRES, controlling for PHE. Parameter $\theta_2$ represents the direct effect of BMI on PHE, and $\theta_3$ represents the causal effect of PHE on PRES. Also included are the posterior distributions for two nonlinear functions of the above parameters, namely $\theta_2 \theta_3$, which represents the indirect component of the effect of BMI on PRES, mediated by PHE, and $\theta_{TOT} \equiv \theta_1+\theta_2 \theta_3$, which represents the total effect of BMI on PRES. From a substantive point of view, these results can be interpreted to provide evidence of a causal effect of the body mass index on blood pressure and phenylalanine concentration, but no evidence that this latter influences blood pressure.

The second stage of our analysis embraced variables BMI, PHE, and PRES. Our assumptions in this analysis are depicted in Figure 2b, where the effect of BMI on PRES has two putative components: a direct one and an indirect component mediated by PHE. We analyzed the data by using model (5.1), with $X_1$, $X_2$ and $Y$ representing log BMI, log PHE, and PRES, respectively. We used a set of 98 instruments, $Z_1, \ldots , Z_{98}$, partitioned into a subset $I_1 \equiv (Z_1, \ldots, Z_{80})$ consisting of 80 BMI-associated instrumental SNPs (the same as in the preceding part of the analysis), and a subset $I_2 \equiv (Z_{81}, \ldots , Z_{98})$, consisting of 18 instruments associated with PHE but not BMI. We assumed almost all the parameters to be a priori uniformly distributed. We sampled the model posterior distribution by running six Markov chains, of 100 000 iterations each, with initial values spanning the approximate 95% confidence intervals for $\theta_2$ and for the quantity $\theta_1 +\theta_2 \theta_3$, as obtained by a traditional MR analysis. We checked convergence of the six chains to the same posterior. The second half of each chain was used to approximate the posterior means and credible intervals for the parameters of interest. Figure 4 shows the marginal posterior distributions for the main quantities of interest. One of the plots shows the posterior distribution for the total causal effect of log BMI on PRES, $\theta_{TOT} \equiv \theta_1+\theta_2\theta_3$. Figure 4 suggests that BMI might exert a causal effect on both PHE and PRES, although there appears to be little evidence of an effect of PHE on PRES. These results discredit the hypothesis of PHE acting as a mediator of the deleterious effect of body mass on blood pressure. The total effect of log BMI on PRES, represented by parameter $\theta_{TOT}$, was re-estimated in the traditional way, by using the instruments contained in $I_1$. This yielded an estimated total effect of 32.3, and a 95% confidence interval of 19.1–46.6, which corresponds to a lower uncertainty relative to the estimate obtained by our method.

In consideration of the relatively small size of the sample, and of the cross-sectional nature of the study, the results of our analysis deserve future independent validation.

## 7. Discussion

Thanks to its holistic approach to uncertainty, a Bayesian approach to MR may represent a safeguard from over-optimistic conclusions. The results of our simulation study are consistent with this expectation, while also suggesting that our method behaves well in the presence of moderate LD between the variants—a welcome feature when the choice of the instruments is confined to a narrow region of DNA.

Much work remains to be done. It might be interesting to assess the extent to which our approach can mimic existing frequentist methods, such as the one proposed by Kang *and others* (2016) and further elaborated by Windmeijer *and others* (2016), where LASSO-type procedures are used to identify the valid instruments from within a set of candidate variables.

A variety of future developments of the approach are envisaged. One of these is to incorporate advances in Bayesian sparsity modeling, for example, in relation to the design of shrinkage priors that deal with high-dimensional vectors of possibly correlated instruments. Of equal importance is to extend the method to deal with nonlinearities and selection effects, and perhaps to incorporate principles of Bayesian model averaging. Such efforts will encounter theoretical difficulties, such as problems of collapsibility of the causal effect parameters. Finally, we may use our framework in a simulation mode, for generating extended datasets from limited data, for purposes of power calculation.

## 8. Software

R software to implement analyses by means of the proposed method is available from Github (https://github.com/carloberzuini/BMR).
